# Simultaneous Quantification of Multiple Immune Checkpoint Interactions in Melanoma

**DOI:** 10.3390/jcm15155771

**Published:** 2026-07-23

**Authors:** Cristina Cacho-Navas, Laura Camacho, Jon Agüero, Baterdene Batmunkh, José María Gracia, Carlos E. de Andrea, Salvador Martín-Algarra, Markel Rementeria, James Miles, Juan Gumuzio, Fernando Aguirre, Peter J. Parker, Véronique Calleja

**Affiliations:** 1 HAWK Biosystems (FASTBASE Solutions S.L.) 612 Astondo Bidea, Science and Technology Park of Bizkaia, 48160 Derio, Spain; cristina.cacho@hawkbiosystems.com (C.C.-N.); laura.camacho@hawkbiosystems.com (L.C.); jon.aguero@hawkbiosystems.com (J.A.); baterdene.batmunkh@hawkbiosystems.com (B.B.); jose.gracia@hawkbiosystems.com (J.M.G.); markel.rementeria@hawkbiosystems.com (M.R.); james.miles@hawkbiosystems.com (J.M.); juan.gumuzio@hawkbiosystems.com (J.G.); fernando.aguirre@hawkbiosystems.com (F.A.); 2 Facultad de Medicina, Departamento de Patología, Anatomía y Fisiología, Clínica Universidad de Navarra (CUN), 31008 Pamplona, Spain; ceandrea@unav.es; 3 Facultad de Medicina, Departamento de Oncología, Clínica Universidad de Navarra (CUN), 31008 Pamplona, Spain; smalgarra@unav.es; 4 School of Cancer and Pharmaceutical Sciences, Comprehensive Cancer Centre, King’s College London, London SE1 1UL, UK; peter.parker@crick.ac.uk; 5 The Francis Crick Institute, London NW1 1AT, UK

**Keywords:** functional proteomics, spatial biology, QF-Pro^®^, FRET-FLIM, immuno-oncology, precision medicine

## Abstract

**Background/Objectives:** Immune checkpoint inhibitors (ICIs) have transformed the therapeutic landscape of advanced malignancies. However, only a subset of patients respond to treatment. Recent efforts have focused on the identification of novel biomarkers that capture the dynamic and functional state of the tumour immune microenvironment, yet their clinical translation has remained challenging. **Methods:** To address these limitations, we applied amplified FRET-FLIM (QF-Pro^®^) technology to spatially resolve and quantitatively assess functional immune checkpoint interactions directly in cells and FFPE tissue and tumour samples. **Results:** Using this approach, we demonstrated that PD-1/PD-L1, CTLA-4/CD80, TIGIT/CD155, and LAG-3/MHC-II interactions can be robustly quantified in routine FFPE patient samples from multiple tumour types. Furthermore, in a proof-of-concept study in a melanoma cohort treated with immune checkpoint inhibitors (ICIs) targeting PD-1 or CTLA-4, co-analysis of all four immune checkpoints suggested patterns of concomitant engagement. Notably, patients with high PD-1/PD-L1 interaction levels tended to also exhibit elevated CTLA-4/CD80 interactions. Survival analysis further showed that high LAG-3/MHC-II interaction status was associated with a trend toward improved overall survival even when corrected for tumour stage, irrespective of the specific ICI regimen administered. **Conclusions:** Although prospective validation in larger independent cohorts would be critical to establish clinical relevance, these findings support the exploration of LAG-3 engagement as a potential biomarker in melanoma immunotherapy, and more broadly highlight immune checkpoint interaction profiling as a promising avenue to examine in patient stratification.

## 1. Introduction

Immune checkpoints (ICPs) are essential regulatory pathways that maintain immune homeostasis and prevent excessive immune activation [1]. In cancer, tumour cells evade immune surveillance exploiting ICP pathways to suppress anti-tumour immunity [2,3]. Immune checkpoint blockade (ICB) targeting PD-1/PD-L1 (nivolumab, pembrolizumab) or CTLA-4 (ipilimumab) has transformed cancer treatment by restoring anti-tumour immune response [4]. Nevertheless, many patients do not respond, and treatment is frequently associated with severe immune-related adverse events (irAEs) [5,6]. This is particularly important in combination treatments like anti-PD-1 with anti-CTLA-4 [7] or the newly approved anti-LAG-3 (relatlimab) in metastatic melanoma, where the improved response is accompanied by about 60% and 20% respectively of grade 3–4 side effects [8]. Therefore, it is critical to identify the patients who will respond to treatment. To do so, companion and complementary diagnostics tests are presently used. They encompass PD-1 and PD-L1 expression, tumour mutational burden (TMB) or T-cell infiltration among others [9]. However, patient stratification based on these biomarkers alone has remained suboptimal, underscoring the need for more robust predictive markers. Although novel biomarkers are under investigation [10], none directly assess checkpoint engagement which is the primary molecular event targeted by immune checkpoint blockade.

Biomarkers assessing the functional state of ICP would be expected to better reflect the actual immune status of the tumour microenvironment (TME) and the potential benefit of a specific intervention. This was previously demonstrated for PD-1/PD-L1 in non-small-cell lung cancer (NSCLC), where it was found that the stratification power associated with PD-1/PD-L1 engagement status in patient biopsies showed a far superior predictive value to ICP treatment, compared to the routinely employed PD-L1 TPS/CPS score [11]. These findings motivate the analysis of a broader panel of ICPs, both to understand the patterns of exclusive or coincident engagement elicited by tumours and to provide a framework in which to establish a molecular pathology applicable in the clinic.

Here, we show that PD-1/PD-L1, CTLA-4/CD80, TIGIT/CD155 and LAG-3/MHC-II immune checkpoints interaction states can be quantified in cells and in FFPE tissue samples from multiple tumour types (NSCLC, Breast and Melanoma). While PD-1/PD-L1 and CTLA-4/CD80 interactions had previously been measured in non-small-cell lung cancer [11,12], the quantification of TIGIT/CD155 and LAG-3/MHC-II interactions had not been explored in prior studies. Among the TIGIT ligands involved in immunosuppression which include CD112 (Nectin-2) and CD113 (Nectin-3), CD155 (PVR) was selected for its highest binding affinity, its broad expression on tumour cells and APCs, and its established role as the primary mediator of TIGIT-dependent immunosuppression [13]. Similarly, although LAG-3 engages additional partners such as FGL1, LSECtin or Galectin-3, MHC-II was chosen as the canonical highest-affinity ligand and the best-characterised mediator of LAG-3-driven T-cell immunosuppression [14]. Together, these considerations informed the design of a multi-checkpoint interaction profiling approach, which we applied here across tumour types and in a melanoma ICI-treated cohort to explore its feasibility and potential relevance as a tool for biomarker discovery.

## 2. Materials and Methods

### 2.1. Primary Antibodies

The primary antibody list is presented in Supplementary Table S1.

### 2.2. Secondary Reagents

QF-Pro^®^ reagent kits (HAWK Biosystems, Derio, Spain) contain all reagents needed to perform the labelling process. Briefly, the reagents provided within the kit include peroxidase suppressor, blocking and washing buffers, fluorescent (ATTO488 (ATTO-TEC GmbH, Siegen, Germany) and AlexaFluor594 (Thermo Fisher Scientific, Waltham, MA, USA)) labelled secondary anti-mouse and anti-rabbit antibodies, amplification reagents and mounting medium.

### 2.3. Melanoma Cohort Inclusion Criteria

Patients diagnosed with advanced melanoma (from Navarra University Hospital (CUN)) treated with systemic immunotherapy based on Nivolumab or a combination of Nivolumab with Ipilimumab, as well as patients treated with Ipilimumab only, were included in the cohort. Benefit from treatment was measured at 12 months by RECIST criteria as above. For each patient, a FFPE sample from the initial tumour resection was obtained. Whole slides from the FFPE tissue block were used (Lopez-Janeiro, A. et al. 2026 [15]).

### 2.4. FFPE-Tissue and Tumour Samples

Formalin-fixed, paraffin-embedded (FFPE) sections of 3–5 mm thickness were obtained from diagnostic biopsies, tumour resections or healthy tissues. Melanoma samples were provided by the Pathology Department of Clínica Universidad de Navarra (CUN), Pamplona, Spain. FFPE tissue samples were obtained with informed patients’ consent and retrieved from the pathology files of the Clínica Universidad de Navarra (protocol 111/2010). Tonsil blocks and TMA slides of breast tumours were purchased from AMS Biotechnology (AMSBIO), Abingdon, UK. FFPE samples were stained with H&E following standard protocol.

### 2.5. QF-Pro^®^ (Quantifying Function in Proteins) Assay

Before incubation with QF-Pro^®^ reagents, pre-treatment processes of deparaffination, rehydration and epitope retrieval were performed using a Dako PT-Link (Dako, Glostrup, Denmark). Samples were then treated following the QF-Pro^®^ labelling protocol. Briefly, samples were incubated with endogenous peroxidase suppressor and then with blocking buffer to prevent non-specific signal. Samples were incubated with primary antibodies overnight at 4 °C and then with donor and acceptor secondary reagents at room temperature (RT) for two hours. Amplification reagent was applied to Donor–Acceptor slides for 20 min and mounting medium was applied. Slides were stored until ready for fluorescence lifetime imaging microscopy analysis. A detailed protocol for QF-Pro^®^ assays can be found in our previous publications [11,16].

### 2.6. Acquisition, Analysis and Quantification

Imaging and quantification of the immune checkpoint interaction states were performed using Violet 3.0. (Fluorescence Lifetime Imaging Microscope), controlled by QF-Pro^®^ software (HAWK Biosystems, Derio, Spain). Violet 3.0 and QF-Pro^®^ software automatically measure the decay of the lifetime of the donor fluorophore in the absence and presence of the acceptor (FRET-FLIM) to calculate interaction states (QF-Pro^®^ scores). Violet 3.0 and QF-Pro^®^ software allow for the spatial quantification of proteomic functional events across the sample. A decrease in donor lifetime (τD) in the presence of the acceptor chromophore (τDA) is indicative of resonance energy transfer. FRET efficiency (Eff % or QF-Pro^®^ score) values were calculated using the following equation, where τD and τDA are the lifetimes of the donor in the absence and presence of the acceptor, respectively:

$$\mathrm{Eff}\;\left(\%\right)=[1-(\frac{\tau DA}{\tau D})]\times 100.$$

### 2.7. Detailed Statistical Analysis

Box-and-whisker plots were generated using GraphPad Prism 10. Here, the boxes represent the 25–75% range of the data and the whiskers represent the minimum and maximum values. Melanoma patients were ranked in order of their mean FRET efficiencies (QF-Pro^®^ scores, i.e., interaction status) and split into two groups using the webtool, Cutoff Finder (version 1.0.0), to define objectively a cutoff point for survival analysis. The tool, described by Budczies et al., 2012, utilises R scripts [17]. The log-rank (Mantel–Cox) test was carried out to determine significant differences between the groups. Kaplan–Meier curves were then plotted using GraphPad Prism 10. The hazard ratio and *p* values were also calculated using GraphPad Prism 10. Correlation analyses were performed using the Pearson correlation test on continuous variables, and corresponding r values and two-tailed *p* values were generated automatically by the software. No correction for multiple comparisons was applied, given the exploratory and hypothesis-generating nature of the study. Applying conservative corrections such as Bonferroni in an already underpowered small cohort would substantially increase the risk of false negatives and is not standard practice in proof-of-concept studies. All reported *p*-values should therefore be interpreted as exploratory findings requiring independent replication.

Correlation matrix was exported directly from Prism 10 for presentation. Statistical significance was defined as *p* < 0.05.

Forest plot analyses were performed using GraphPad Prism 10 to visualise univariate and bivariate Cox regression analysis of the clinical and FRET-FLIM interaction parameters. Hazard ratios (HRs) and corresponding 95% confidence intervals for each variable were plotted as point estimates with horizontal confidence interval bars. A reference HR = 1 was used to indicate the null effect. Statistical calculations and graphical outputs were generated directly within Prism 10.

### 2.8. Cell Cultures and Sensitivity Assays

The commercially validated Promega Blockade Bioassay kits, originally designed to measure the antibody blockade of PD-1/PD-L1 and TIGIT/CD155 interaction by luminescence, were adapted for performing QF-Pro^®^ protocol. Cells were thawed and directly used in this assay following the recommendations of the manufacturer. PD-L1 or CD155-expressing CHO-K1 cells were seeded onto 96 well plates and were incubated at 37 °C with 5% CO_2_ for 16 h. PD-1 or TIGIT-expressing Jurkat cells together with the blocking antibodies (against PD-1 or TIGIT respectively) were added to the CHO-K1cells. The co-cultures were incubated for 20 h at 37 °C with 5% CO_2_. The unbound cells were removed by washing with PBS before being fixed with 4% PFA for 15 min. After fixation, cells were washed with PBS and stored at 4 °C until labelling. QF-Pro^®^ labelling was performed as described for FFPE-samples.

## 3. Results

**Figure 1:**
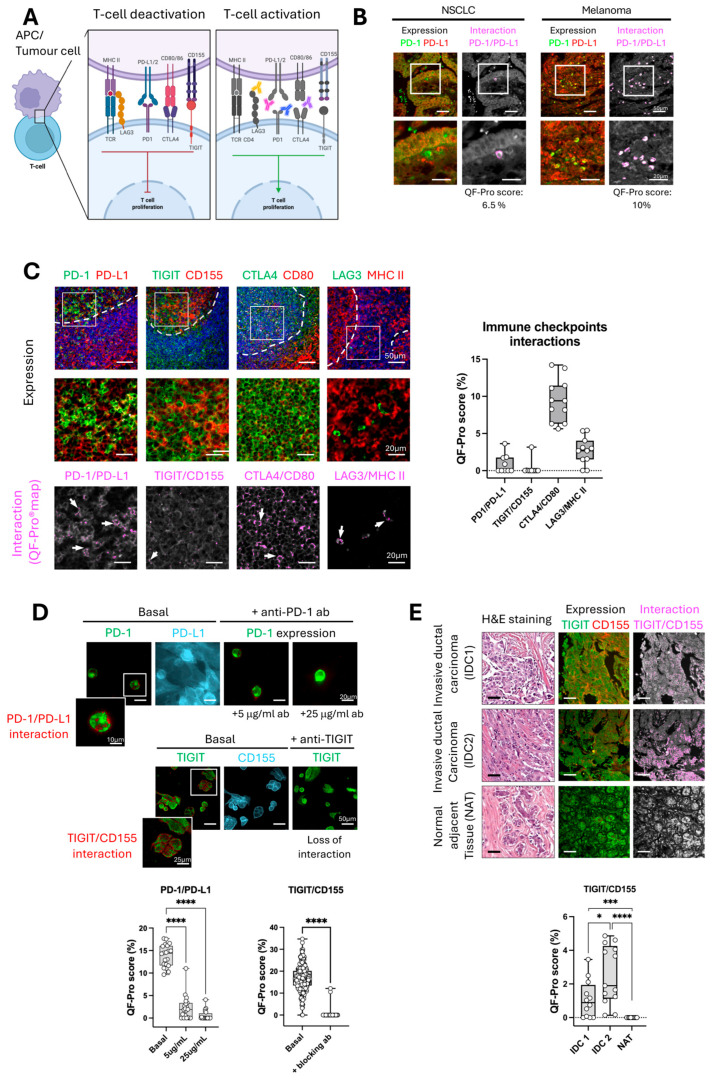
Quantitative measurement of immune checkpoint complex engagement across multiple models. (**A**) Schematic representation of immune checkpoint receptor–ligand complexes and their corresponding therapeutic monoclonal antibodies. (**B**) PD-1/PD-L1 expression (green and red) and interaction (magenta) in NSCLC and melanoma tissues. Interaction maps are overlaid with PD-1 expression (grey), detectable on infiltrating immune cells in both tumour types. * *p* < 0.05, *** *p* < 0.001, **** *p* ≤ 0.0001. (**C**) Human FFPE tonsil tissue labelled to quantify four immune checkpoint interactions as indicated. Donor (green), acceptor (red), and nuclei (blue) signals are shown for each checkpoint pair. Spatial localisation of immune checkpoint engagement (QF-Pro maps, magenta) is overlaid with donor expression (grey). Box-and-whisker plots display quantitative interaction scores (QF-Pro^®^ scores), with each dot corresponding to a region of interest. (**D**) Validation of PD-1/PD-L1 and TIGIT/CD155 interactions in cell-based co-culture assays using Promega Blockade Bioassay Kits (Promega Corporation, Madison, WI, USA), including blocking antibody controls. Biomarker expression is shown in green or blue as indicated, while interactions are shown in red and quantified in box-and-whisker plots, with each dot representing a single cell. (**E**) Invasive ductal carcinoma and adjacent tissues labelled for TIGIT/CD155. TIGIT (green), CD155 (red), and nuclei (blue) expression are shown, with TIGIT/CD155 interaction localization (QF-Pro^®^ map, magenta) overlaid on donor expression (grey).

Here, an amplified FRET-FLIM technology (QF-Pro^®^) was employed to determine whether in situ engagement of key immune checkpoints (PD-1/PD-L1, CTLA-4/CD80, TIGIT/CD155 and LAG-3/MHC-II) could be quantified across cells types and multiple tissue contexts, with the potential to generate clinically relevant insights for immune checkpoint inhibitor therapy (Figure 1A).

The amplified FRET signal enables highly accurate quantification of proteins’ functional states within complex biological microenvironments, including FFPE tumour samples (Supplementary Figure S1, Section 2). Primary commercial antibodies to these checkpoints were selected based on (i) epitope recognition within the extracellular domains of the corresponding ligands or receptors, and (ii) prior validation by the suppliers for use in FFPE samples (Supplementary Table S1).

### 3.1. PD-1/PD-L1 Immune Checkpoint Interaction Can Be Detected in Infiltrated Immune Cells in NSCLC and Melanoma Tissue

We previously exemplified the predictive value of monitoring PD-1/PD-L1 interactions in assessing response to immune checkpoint inhibitor (ICI) therapy in NSCLC [11]. Associated with the work to extend this approach to a broader collection of checkpoints, we first developed a more comprehensive spatial mapping tool to improve interpretation of findings.

The spatial localization of PD-1/PD-L1 interactions was examined in NSCLC and melanoma tumour tissues, providing additional contextual insight into these interactions. QF-Pro^®^ maps (magenta) revealed that PD-1/PD-L1 interactions occur on infiltrating lymphocytes within the tumour microenvironment (TME) in both tissue types (Figure 1B). Interaction levels were quantified using QF-Pro^®^ scores, calculated from signals within a defined region of interest (ROI), indicated by the white square and corresponding zoomed image.

### 3.2. Immune Checkpoint Interaction Detection in FFPE Tonsil Tissue

With a mapping tool in hand we first sought to validate the four ICP interactions in a tissue model. The engagement of PD-1/PD-L1, CTLA-4/CD80, TIGIT/CD155, and LAG-3/MHC-II was therefore measured in FFPE tissue sections of human tonsil tissue (Figure 1C). As a secondary lymphoid organ, the tonsil plays a critical role in early immune priming and immune responses, where multiple immune cell populations interact closely with antigen-presenting cells [18]. These characteristics make tonsil tissue an effective model for assessing immune checkpoint interactions in situ.

Interaction quantification (QF-Pro^®^ scores) and spatial localisation (QF-Pro^®^ maps; magenta) were successfully determined for three of the four immune checkpoints. Notably, the TIGIT/CD155 interaction was not detectable in tonsil tissue (one outlier), as evidenced by both the interaction map image and the box-and-whisker plot (Figure 1C righthand panel). The expression of the corresponding immune receptors and ligands (green and red) was however detected in DAPI-stained tissue sections for all the checkpoints. The data revealed variability in interaction levels as well as distinct spatial localisation patterns among the different immune checkpoints within the same tissue. Importantly, the specific interaction patterns (or lack thereof) observed in the interaction maps are distinct from simple colocalisation of checkpoint protein expression (green and red), supporting the importance of direct interaction mapping. This is particularly evident in the labelling of TIGIT/CD155 where robust expression of both proteins is observed within the same region (see the zoomed middle panel of the boxed area), yet no interaction is visible on the QF-Pro^®^ map (lower panel).

### 3.3. PD-1/PD-L1 and TIGIT/CD155 Interactions Measured by QF-Pro^®^ Are Validated in Co-Culture Models

As all immune checkpoint pairs except TIGIT/CD155 showed detectable engagement in tonsil tissue, we sought to validate the TIGIT complex readout using a cell line model. To this end we adapted the Promega Blockade Bioassay kits to enable measurement of TIGIT/CD155 and, as a positive control, PD-1/PD-L1 interactions using the QF-Pro^®^ platform (see Section 2). These ICP interactions were assessed in co-cultures of CHO-K1 cells engineered to express either human PD-L1 or CD155 and Jurkat cells stably expressing human PD-1 or TIGIT (Promega) (Figure 1D). PD-1/PD-L1 and TIGIT/CD155 interactions (red) were detected in overlay images with donor protein expression (PD-1 and TIGIT, as indicated shown in green). Notably, these interactions were abolished upon addition of blocking antibodies against PD-1 or TIGIT (respectively) prior to fixation. Quantification of interaction signals and their loss following interfering antibody-mediated blockade is shown in the box-and-whisker plots (Figure 1D lower panels), demonstrating the specificity of these immune checkpoint engagements in engineered in-cell systems.

### 3.4. TIGIT/CD155 Interaction Is Observed in Invasive Ductal Carcinoma Tissue

Because the interaction between TIGIT and CD155 was detectable in the cell co-culture system, despite not being observed in tonsil, we anticipated that TIGIT/CD155 interaction would be detectable in a tissue where it is more highly enriched. In invasive breast cancer (invasive ductal carcinoma, IDC), upregulation of TIGIT expression has been associated with poor prognosis, suggesting a prominent role for TIGIT/CD155 in the negative regulation of immune responses within the TME in these tumours [19]. Therefore, TIGIT/CD155 interaction was assessed in two IDC samples and normal adjacent tissue (NAT) from a tumour microarray (TMA) (Figure 1E). Quantifiable TIGIT/CD155 interaction was detected with variability in the tumour samples but no interaction was detected in the non-tumoral tissue (interaction maps and box-and-whisker plot). These findings indicate that the TIGIT/CD155 complex can be quantified in IDC, providing direct information on the status of this interaction within these tumours.

### 3.5. Analysis of ICP Interactions in a Cohort of Advanced Melanoma Patients Treated with Immune Checkpoint Inhibitors as Second-Line Treatment

Having validated the ability of QF-Pro^®^ and the selected antibody pairs to detect PD-1/PD-L1, CTLA-4/CD80, TIGIT/CD155, and LAG-3/MHC-II immune checkpoint interactions in FFPE tissue and tumour samples, we next investigated whether their combined analysis within individual patient samples could reveal patterns of checkpoint engagement. Specifically, the parallel assessment of ICP interaction was performed to determine whether these pathways are activated concomitantly or operate in a mutually exclusive manner within tumours. To address this we conducted a study in a cohort of melanoma patients (n = 30).

An overview of the clinical characteristics of the melanoma cohort is provided in Supplementary Figure S2 and Supplementary Table S2 and the patient inclusion criteria can be found in Section 2. The patients received a range of immune checkpoint inhibitor regimens: (i) anti-PD-1 monotherapy (pembrolizumab or nivolumab; n = 8), (ii) anti-CTLA-4 monotherapy (ipilimumab; n = 14), and (iii) combined anti-PD-1 and anti-CTLA-4 therapy (nivolumab plus ipilimumab; n = 8). A Cox regression analysis of OS presented as a forest plot (Supplementary Figure S2A) was performed to evaluate the prognostic impact of the clinical characteristics. Hazard ratio (HR) analysis revealed no statistically significant differences in mortality risk across the clinical subgroups. However, a trend was observed in which patients with metastatic disease exhibited a reduced median OS compared with non-metastatic patients (21 vs. 32 months, respectively; Supplementary Figure S2B). Consistent with this finding, patients with metastatic disease also showed a lower clinical benefit at 12 months compared with those with primary, non-metastatic tumours (28% vs. 42%, respectively; Supplementary Figure S2C). Overall, these analyses suggest that none of the evaluated clinical variables introduced significant bias that could substantially influence the observed results.

**Figure 2:**
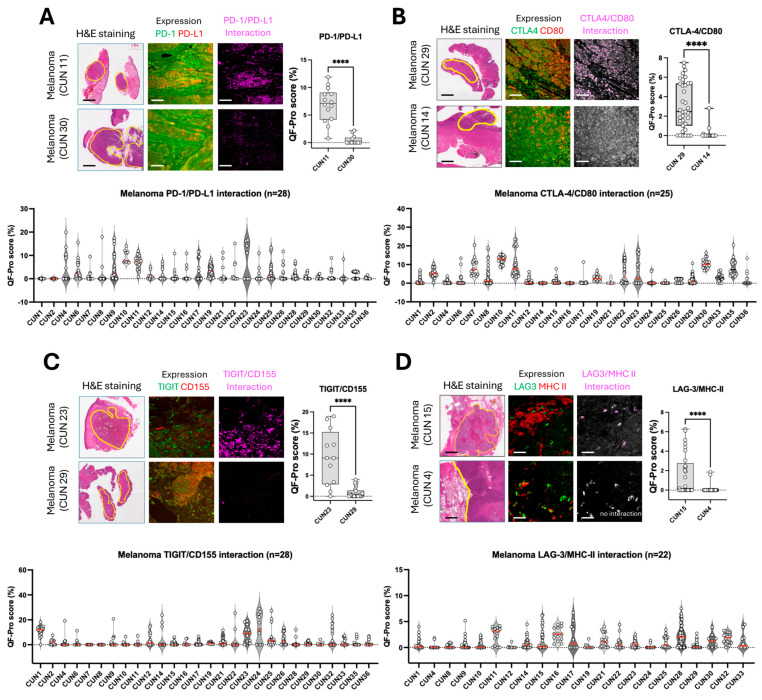
Spatial analysis of ICP engagement in the TME and heterogeneity across the melanoma cohort. (**A**–**D**) Expression (green and red) and interaction (magenta) of immune checkpoint receptors and ligands are detected using QF-Pro^®^ mapping. Representative patient samples illustrate PD-1/PD-L1 (**A**), CTLA-4/CD80 (**B**), TIGIT/CD155 (**C**), and LAG-3/MHC-II (**D**) interactions, localisation and heterogeneity. The images of the ICP were all taken from the delineate tumour regions (yellow; H&E images). Box-and-whisker plots depict variability in interaction across multiple fields of interest for the same patient, with each dot representing one field of view. **** *p* ≤ 0.0001. Violin plots present interaction heterogeneity (QF-Pro^®^ scores) across all patients for the four checkpoints. Each dot represents a region of interest acquired for each patient sample. The median QF-Pro^®^ interaction scores are indicated by red bars.

LAG-3/MHC-II, CTLA-4/CD80, TIGIT/CD155, and PD-1/PD-L1 interactions were quantified and imaged within the tumour regions in the melanoma cohort. The identification of the tumour region was done by an experienced pathologist (C.E.A., Clinica Universidad de Navarra) who delineated tumour regions on H&E-stained sections (yellow outline; Figure 2A–D) prior to and independently of QF-Pro^®^ interaction analysis and was blinded to interaction outcome data. A minimum of eight regions of interest (ROIs) per patient depending on the size of the region (ROI number 8-61, median 18) were acquired within the delineated tumour areas and the mean QF-Pro^®^ score across all ROIs was used as the per-patient value for statistical analyses. Representative images were chosen to illustrate patients’ samples with higher ICP interaction versus patients’ samples with lower interaction for each of the four checkpoints. Intensity images show the expression of immune checkpoint receptors (LAG-3, CTLA-4, TIGIT, and PD-1; green) and their corresponding ligands (MHC-II, CD80, CD155, and PD-L1; red) in the regions of interest (ROI) shown. QF-Pro^®^ maps (magenta) depict the spatial localisation of each ICP interaction within the tumour ROI. The distribution of QF-Pro^®^ scores measured at each ROI (individual dots) are presented in the associated box-and-whisker plots, highlighting the interaction heterogeneity and strong statistical difference between these patient examples (*p* < 0.0001). The distribution of the immune checkpoint interactions (QF-Pro^®^ scores) across the melanoma patient cohort is presented on the violin plots (below the images), illustrating further the intra- and inter-patient heterogeneity of immune checkpoint interactions.

Notably, analysis of the LAG-3/MHC-II localisation in the TME (Figure 2D) shows that while both the patients shown present comparable expression of LAG-3 in infiltrated immune cells (see expression panels; green), only patient CUN15 shows interaction (upper interaction panel; magenta). This data further highlights the importance of interaction quantification over protein colocalisation, even when proteins are present within the same spatially defined regions.

### 3.6. Co-Engagement Analysis of Immune Checkpoints in the Tumour Microenvironment

The determination of the interaction status of the four ICPs in each patient within this cohort, offered the opportunity to analyse whether the concurrent quantification of the four immune checkpoint interactions in the same patient’s samples could inform on co-regulation or mutual exclusivity of the ICP axis engagement in the tumours.

**Figure 3:**
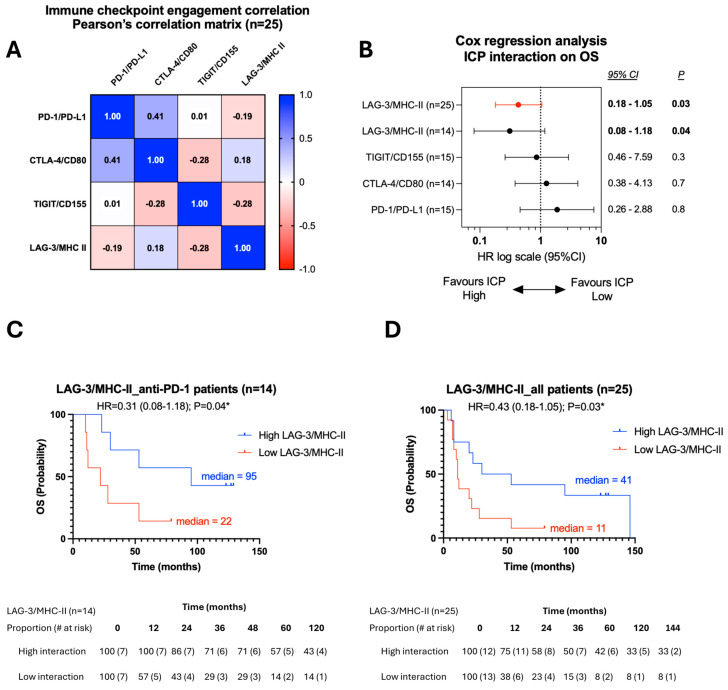
Immune checkpoint interaction profiling and association with overall survival in the melanoma cohort. (**A**) Pearson’s correlation matrix of pairwise ICP interaction scores across all four checkpoint axes (n = 25). Correlation coefficients (r) are displayed within each cell and colour-coded from red (negative) to blue (positive). Only the correlation between PD-1/PD-L1 and CTLA-4/CD80 reached statistical significance (r = 0.41, *p* = 0.04). (**B**) Univariate Cox proportional hazards analysis of overall survival by ICP interaction status. Forest plot presenting hazard ratios (HRs), 95% confidence intervals (CIs), and *p*-values for each ICP axis. Results are shown for the primary anti-PD-1-based cohort and, for LAG-3/MHC-II, additionally for the sensitivity cohort (n = 25). The arrow direction indicates whether high interaction status favours or disfavours survival. (**C**) Kaplan–Meier analysis of OS in patients treated with anti-PD-1-based regimens (n = 14), stratified by high vs. low LAG-3/MHC-II interaction status. Median OS was 95 months (high) vs. 22 months (low); HR = 0.31 (95% CI 0.08–1.18), *p* = 0.04. (**D**) Kaplan–Meier analysis of OS in the sensitivity cohort including patients treated with anti-CTLA-4-based regimens (n = 25), stratified by high vs. low LAG-3/MHC-II interaction status. Median OS was 41 months (high) vs. 11 months (low); HR = 0.43 (95% CI 0.18–1.05), *p* = 0.03. Numbers at risk at each time point are shown in the tables below each Kaplan–Meier curve. Spatial quantification of immune checkpoints interactions in the ICI-treated melanoma cohort. * *p*-value < 0.05.

A Pearsons’ correlation analysis, visualised using a correlation matrix was performed (Figure 3A). Pairwise relationships between the ICP interaction states were assessed by the correlation coefficients (r) shown in the matrix. R ranges from −1 (perfect inverse correlation; in red) to +1 (perfect positive correlation; in blue), with values close to 0 indicating no linear relationship and with the increasing colour intensity corresponding to stronger associations. The data revealed a positive linear correlation between PD-1/PD-L1 and CTLA-4/CD80. The moderate value of the Pearson’s r (r = 0.41; *p* = 0.04) suggested a possible co-engagement of these checkpoint interactions in melanoma, similarly to what had been previously observed in NSCLC [12], though validation in a larger study would be essential to confirm this result. No other correlations could be observed between other checkpoint combinations within this cohort.

### 3.7. LAG-3/MHC-II Engagement Informs on Immune Checkpoint Inhibitor Response in the Melanoma Cohort

Exploiting the simultaneous evaluation of four ICP interactions within this limited cohort, we compared the potential of each ICP to associate with clinical outcome following ICI treatment. To this end, OS of patients stratified into high versus low ICP-interaction groups were examined (Figure 3B). The QF-Pro^®^ scores cutoff values were determined using the online tool “Cutoff finder” [17] (Supplementary Table S3 and Section 2).

Given the markedly inferior overall survival of anti-CTLA-4 monotherapy patients compared with anti-PD-1-based regimens (median OS 8 vs. 41 months, *p* = 0.03; Supplementary Figure S3), and the high rate of non-response (87%) in this subgroup (Supplementary Table S4), these patients were excluded from the primary survival analysis based on their distinct clinical profile, to avoid confounding by treatment-specific failure.

The association between high vs. low interaction states and survival outcomes was evaluated using a univariate Cox proportional hazards analysis. The resulting hazard ratios (HRs) and 95% confidence intervals (CIs) are summarised in a forest plot (Figure 3B), illustrating effect size and direction across all immune checkpoints. Despite the limited cohort size, high LAG-3/MHC-II interaction status showed a trend toward improved overall survival (HR = 0.31, 95% CI 0.08–1.18), corresponding to an approximately 70% reduction in the risk of mortality, though the wide 95% confidence interval reflects the limited sample size. To assess whether this association could be explained by any confounding impact from cohort heterogeneity, metastatic vs. primary tumour (the most relevant clinical variable identified) was included as a covariate in the bivariate Cox model analysis of LAG-3/MHC-II interaction status on OS (Supplementary Figure S4). High LAG-3/MHC-II interaction status remained independently associated with improved OS after adjustment (HR = 0.22, 95% CI 0.04–0.98), while tumour stage did not reach statistical significance (HR = 1.43, 95% CI 0.33–6.40).

In a sensitivity analysis reintroducing the anti-CTLA-4 treated patients (n = 25), high LAG-3/MHC-II interaction status still showed a trend toward improved OS (HR = 0.43, 95% CI 0.18–1.05) showing nearly 60% reduction in the risk of mortality, and a tighter 95% CI as compared to the smaller cohort despite not reaching significance (Figure 3B, red bar). In the bivariate Cox model analysis of this larger cohort corrected for tumour stage the results were consistent with earlier findings, where high LAG-3/MHC-II interaction state remained independently associated with improved OS (HR = 0.33, 95% CI 0.11–0.91) suggesting the finding is robust to cohort composition even in the larger cohort (Supplementary Figure S4).

In contrast to the above, CTLA-4/CD80, TIGIT/CD155 and PD-1/PD-L1 interaction states did not display a consistent directional association with OS, as reflected by the wide 95% confidence interval. The Kaplan–Meier analysis of survival illustrates the association of LAG-3/MHC-II interaction state with the subgroup of patients who responded to anti-PD-1-based treatments (Figure 3C). A high LAG-3/MHC-II interaction was associated with a marked OS benefit compared with low interaction levels (95 vs. 22 months; *p* = 0.04). Incorporating patients treated with ipilimumab (n = 25) into the survival analysis showed that a high LAG-3/MHC-II interaction state continued to identify patients with longer overall survival (41 vs. 11 months; *p* = 0.03; Figure 3D); this association was therefore not restricted to patients receiving anti-PD-1-based regimen.

## 4. Discussion

Immune checkpoint inhibitors have transformed the therapeutic landscape of advanced malignancies; however, a substantial proportion of patients fail to respond, and the PD-L1 (TPS) expression biomarker currently used in clinical practice has shown limited predictive accuracy. This likely stems from its inability to directly inform on functional PD-1/PD-L1 engagement, making it a poor surrogate of the true immune checkpoint inhibitor target status. Conversely, the remarkable predictive value of PD-1/PD-L1 engagement for response to immunotherapy as compared to PD-L1 TPS in NSCLC [11], highlights the importance of exploiting such functional biomarkers to predict patient response to ICIs. Here, we extended previous findings to the analysis of the interaction states of four immune checkpoints: PD-1/PD-L1, CTLA-4/CD80, TIGIT/CD155, and LAG-3/MHC-II, all of which are considered to play a role in tumour immune evasion. Spatial localisation and quantification of these interactions across various settings enabled the assessment of the patterns of multiple engagements in situ, not reported previously.

Enhanced FRET–FLIM-based technology was employed to quantitatively measure these interactions (QF-Pro^®^ scores) and to spatially resolve them (QF-Pro^®^ maps) from cell models to tissue and tumour sections, providing a novel approach to interrogating the tumour immune landscape in FFPE samples. Consistent with prior observations in NSCLC, the sensitivity and specificity of the method enabled the detection of both intra- and inter-patient heterogeneity in checkpoint interactions. Moreover, differences observed between the spatial localisation of ICP interactions, and the corresponding receptor–ligand expression patterns, support the assertion that expression-based analyses do not reliably capture functional immune states. These observations are consistent with previous reports showing that direct quantification of checkpoint engagement provides a more informative indicator of tumour immune status [11]. These findings suggest that spatially resolved interaction mapping could provide an additional layer of information beyond interaction quantifications alone, offering further functional insight into the TME ICP landscape.

Having validated the methodology for the identification of ICP interactions in FFPE tumour tissues, a small cohort of melanoma patients treated with immune checkpoint blockade was used to enable simultaneous analysis of four ICPs per patient and to explore potential associations between their interaction states. Pearson’s correlation analysis performed on this cohort revealed a moderate positive correlation between PD-1/PD-L1 and CTL4/CD80 interactions (r = 0.41; *p* = 0.04) while no significant correlations were observed between any of the other ICP pairs. Given the small sample size and the absence of correction for multiple comparisons, this finding should be considered exploratory and hypothesis-generating. The co-occurrence may reflect the simultaneous infiltration of distinct immune cell populations within the TME, including CTLA-4-expressing cells involved in early T-cell priming and PD-1-expressing exhausted cytotoxic T-cell, although the validation of this finding would require further investigation in a larger cohort to confirm interpretation. Nevertheless, this analysis offers a first proof-of-concept for exploring how these pathways may co-occur or function independently across patients. Should this observation be confirmed, the identification of patients with concomitant high engagement of both axes could inform the rational selection of combination ICI regimen, potentially improving benefit–risk assessment. Alternatively, as this co-regulation may reflect an activated yet autoinhibited immune state within the TME, such tumours may retain sensitivity to reactivation through anti-PD-1 monotherapy alone, as recently suggested in NSCLC [12].

Further analysis was conducted in a subgroup of patients receiving the PD-1-based regimen (n = 14), as a proof-of-concept to explore whether ICP interaction states might be associated with OS. The patients were stratified into high- and low-interaction groups using data-driven cutoffs derived from the web-based application Cutoff finder [17]. Cox proportional hazards analysis suggested a directional association between high LAG-3/MHC-II interaction status and improved OS (HR = 0.31, 95% CI 0.08–1.18), with a trend supported by Kaplan–Meier analysis showing a 73 month difference in median survival between high and low-interaction group (*log rank test p* = 0.04). Given that the confidence interval crosses 1, these finding should however be regarded as exploratory. No comparable association was observed for the other ICP axes.

To assess whether this association could be explained by the confounding bias due to tumour stage, a bivariate Cox model adjusting for primary vs. metastatic tumour was performed. High LAG-3/MHC-II interaction status remained independently associated with improved OS after adjustment (HR = 0.22, 95% CI 0.04–0.98), while tumour stage did not reach statistical significance, suggesting the association is unlikely to be driven by cohort heterogeneity alone.

To further assess the robustness of this finding, a sensitivity analysis extending the cohort to include patients treated with CTLA-4 blockade (n = 25) showed consistent directional association with a 30-month difference in median OS between high and low-interaction groups (HR = 0.33, 95% CI 0.11–0.91, *p* = 0.03). Collectively, these findings suggest that higher LAG-3/MHC-II engagement may be associated with improved OS more consistent with a broader prognostic role rather than a treatment-specific predictive effect. However, without an untreated control arm, a formal distinction between prognostic and predictive value cannot be established and will require prospective validation in larger independent cohorts before any clinical conclusions can be drawn.

The mechanism underlying the association between LAG-3/MHC-II interaction and improved outcomes with anti–PD-1-based therapy may be explained by the fact that it could reflect a “hot” TME with the presence of inhibited but tumour-reactive T-cells. In accordance, LAG-3 expression has been associated with CD8^+^ T-cell infiltration and an inflamed tumour microenvironment in melanoma, and its co-expression with PD-1 marks a therapeutically targetable exhausted T-cell population [20]. Accordingly, the interaction map enabled the spatial identification of LAG-3/MHC-II-positive immune cell infiltrations in melanoma (Figure 2D) as well as PD-1/PD-L1-positive infiltration in examples of melanoma and NSCLC (Figure 1B). Presently, single-agent anti-PD-1 therapy (pembrolizumab or nivolumab) typically remains the standard of care achieving objective response rates of about 40%. However, LAG-3 combination therapies (nivolumab plus relatlimab) have been approved for advance melanoma owing to greater efficacy compared to nivolumab monotherapy (RELATIVITY-047 [21] RELATIVITY-098 [22]), despite being associated with increased toxicity, with higher incidence of immune-related adverse events (irAEs) [22].

Although recent clinical data suggest that LAG-3 expression alone does not reliably predict which patients will benefit from anti-LAG-3 therapy [21], quantification of LAG-3/MHC-II interactions, providing a more direct and robust measure of LAG-3 axis activation, may represent a more informative approach. This hypothesis would merit evaluation in cohorts of patients treated with nivolumab monotherapy or in combination with relatlimab.

While anti-TIGIT-targeted therapies have not received clinical approval, analysing TIGIT/CD155 interaction may offer important insights for drug development and therapeutic application. Although early results with anti-TIGIT agents such as tiragolumab generated initial optimism, outcomes from phase II and III trials have so far failed to demonstrate clinical benefit [23]. As a result, patient stratification has been proposed as a strategy to improve therapeutic precision [24]. In this context, directly assessing TIGIT/CD155 interactions, which more accurately reflect immune checkpoint engagement, could be valuable for identifying patients most likely to benefit from intervention.

While these findings support further investigation into the potential utility of ICP interaction states as biomarkers in melanoma, several limitations of this proof-of-principle study should be acknowledged. The relatively small cohort size (n = 30, with subgroups of n = 14–25 for survival analyses) represents the primary limitation, contributing to wide confidence intervals and reduced statistical power. Data-driven cutoff values obtained using Cutoff Finder may introduce optimisation bias; however, alternative approaches such as median split, quartile-based thresholds carry analogous limitations in small samples without offering the advantage of biological grounding. Together these limitations underscore the exploratory nature of the study and the need for prospective validation in larger independent cohorts to assess the robustness and generalisability of these observations.

## 5. Conclusions

Taken together, these findings underscore the complexity of immune regulation in cancer and support the rationale of parallel assessment of multiple immune checkpoints to better characterise the immune landscape. By capturing the co-occurring or independent regulation of immune checkpoint pathways, such an approach may provide a deeper and more accurate understanding of the mechanisms by which tumours evade immune surveillance. This, in turn, may inform the development of more personalised immunotherapy strategies, offering a framework to optimise treatment selection, anticipate resistance, and potentially reduce immune-related adverse effects. Prospective validation in larger independent cohorts will be essential to determine the predictive and prognostic utility of ICP interaction profiling and to establish its potential role in clinical decision making.

## Data Availability

The data that support the findings of this study are available from the corresponding author upon reasonable request.

## Supplementary Materials

The following supporting information can be downloaded at https://www.mdpi.com/article/10.3390/jcm15155771/s1.
