# Supplementary material for: Simultaneous Quantification of Multiple Immune Checkpoint Interactions in Melanoma
Source: J Clin Med. 2026 Jul 23;15(15):5771. doi: 10.3390/jcm15155771 (PMC13467343; doi:10.3390/jcm15155771)
Supplement: Supplementary file 1 [file jcm-15-05771-s001.zip › jcm-4355179-supplementary.pdf]

Supplementary figures

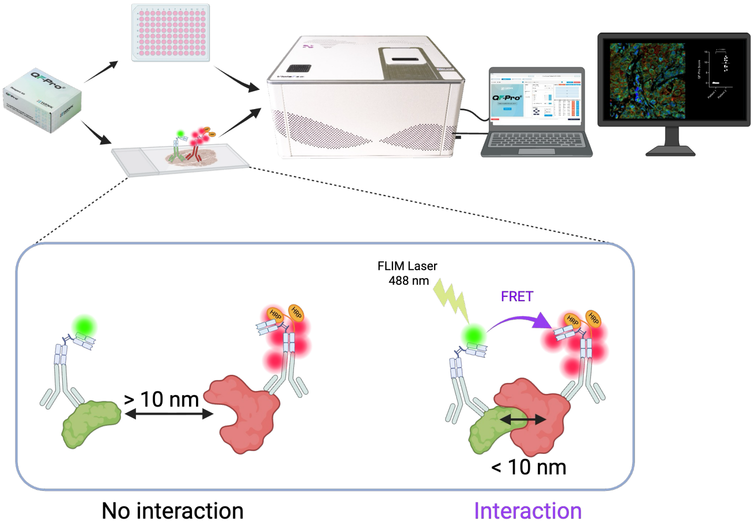

Supplementary Figure S1 Cacho et al.

Supplementary Figure S 1: Quantifying Function in Proteins (QF-Pro®) technology. The technology comprises specific fluorescent secondary reagents, Fluorescence Lifetime Imaging Microscopy (FLIM) platform and dedicated software package for quantification of protein complex formation or post translational modifications in cells or in FFPE tissue samples. The cartoon illustrates the interaction between two proteins recognised by primary and fluorescent secondary antibodies. The interaction is detected by quantifying the transfer or energy (FRET) between the 488nm-laser excited donor (ATTO488) and the amplified acceptor (Alexa Fluor 594). FRET only occurs only when the two proteins are at a binding distance (< 10 nm) and is rapidly lost when the distance between the proteins increases.

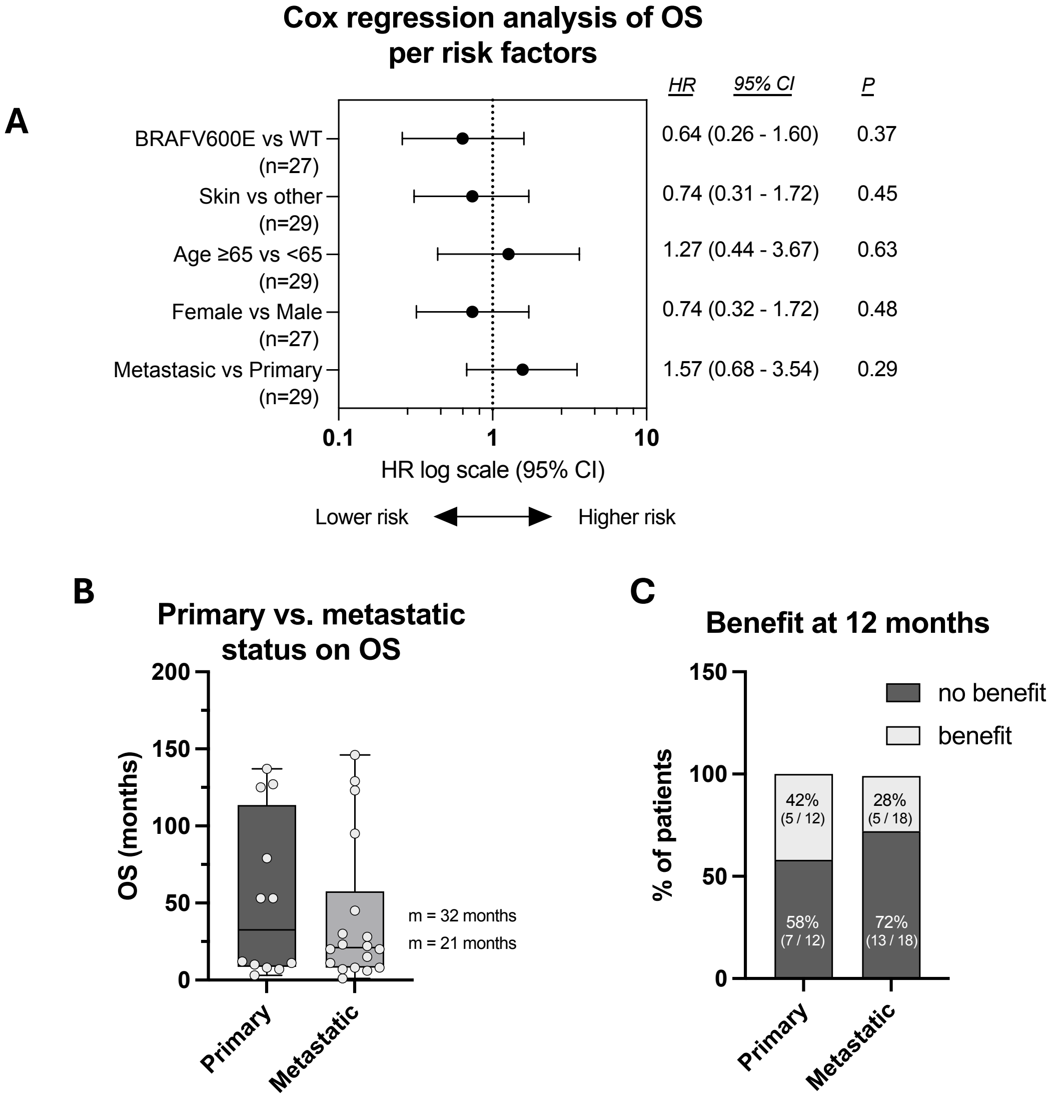

Supplementary Figure S2 Cacho et al.

Supplementary Figure S2: Clinical characterisation of the melanoma cohort. (A) Univariate Cox proportional hazards analysis of overall survival (OS) per clinical risk factors. Forest plot presenting hazard ratios (HR) and 95% confidence intervals (CI) on a log scale, with P-values. Each variable was tested independently. n values are indicated per variable. No variable reached statistical significance. HR values lower than 1 indicate lower risk of death. **(B)** OS distribution by tumour stage (primary vs. metastatic). Box plots show individual patient OS values; median OS

was 32 months for primary and 21 months for metastatic tumours. **(C)** Clinical benefit at 12 months by tumour stage. Stacked bar charts show the proportion of patients with and without clinical benefit at 12 months in primary (n=12) and metastatic (n=18) subgroups. Clinical benefit was observed in 58% (7/12) of primary and 72% (13/18) of metastatic patients.

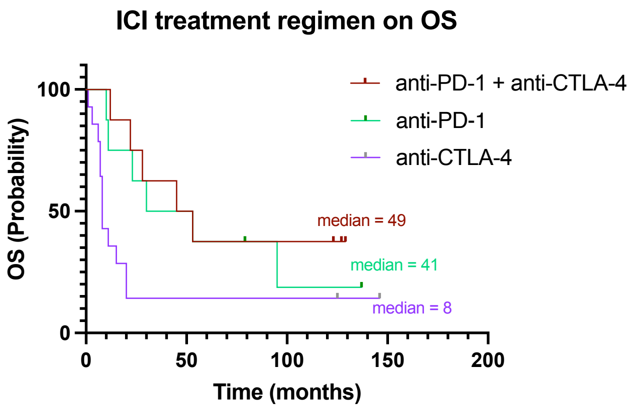

Survival proportion (Number at risk)	Time (months) 0	12	24	60	120	144
anti-PD-1/anti-CTLA-4	100 (8)	87 (8)	75 (7)	38 (4)	38 (4)	38 (1)
anti-PD-1	100 (8)	75 (7)	62 (6)	38 (4)	19 (2)	19 (1)
anti-CTLA-4	100 (14)	36 (6)	14 (4)	14 (4)	14 (4)	14 (2)

Supplementary Figure S3 Cacho *et al.*

Supplementary Figure S 3. Kaplan-Meier analysis of the melanoma patients survival dependent upon immune checkpoint inhibitors treatment. Median survival for anti-CTLA4 compared with anti-PD-1 monotherapy (8 vs. 41 months; P = 0.02) and anti-CTLA4 with anti-PD-1 plus anti-CTLA-4 combination (8 vs. 49 months; P = 0.006). The table presents the survival proportion and the number at risk in brackets, over a period of 144 months.

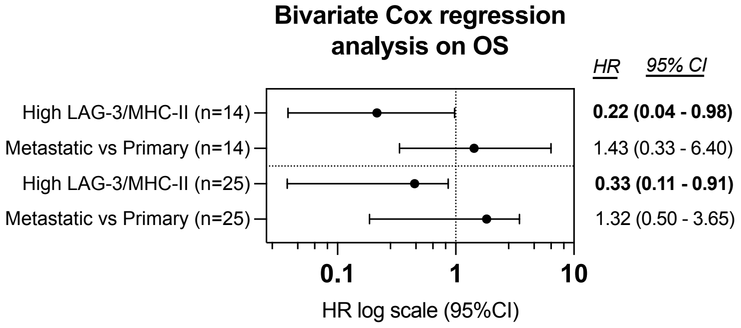

Supplementary Figure S4 Cacho *et al.*

Supplementary Figure S4: Bivariate Cox regression analysis of LAG-3/MHC-II interaction status on OS adjusted for tumour stage. Forest plot presenting hazard ratio (HR) and 95% confidence interval on a log scale. Upper panel (n=14): primary analysis restricted to patients receiving anti-PD-1-based regimens, excluding anti-CTLA-4 monotherapy patients. Lower panel (n=25): sensitivity analysis including patients treated with anti-CTLA-4 based regimen. In both models tumour stage (metastatic vs primary) was included as a covariate. Bold values indicate that the 95% CI does not cross 1. HR values lower than 1 indicate a lower risk of death associated with the factor.

Supplementary Tables

Supplementary Table S1: Primary antibody selection.

Antibody	Catalogue Number
Anti-PD-1 NAT105 mouse mAb (Recombinant)	ab234444
Anti-PD-L1 28-8 rabbit mAb	ab205921
Anti-TIGIT 4A10 mouse mAb	RF16054
Anti-CD155 rabbit mAb	NBP2-78711
Anti-LAG3 mouse mAb	ab307517
Anti-MHC II rabbit mAb	ab92511
Anti-CTLA4 mouse mAb	ABIN6992223
Anti-CD80 rabbit polyclonal Ab	MBS2522916
TIGIT blocking antibody	Promega J2051
PD-1 blocking antibody	Promega J1201

Supplementary Table S2: General clinical data of the melanoma cohort

**Cohort Size and Sex**	
Total patients	30
Female	10
Male	18
Unknown / missing	2
**Age**	
<65	23
≥65	7
median (years)	52
**Tumour stage**	
Primary tumour	12
Metastatic disease	18
**BRAF V600E Status**	
Positive (n=6 treated with BRAF inh±MEK inh before ICI)	8
Negative	20
N/A / Unknown	2
**Tumour Location**	
Skin	17
Other location	13
**Sample Type**	
Biopsy	7
Resection	23
**ICI Treatment Regimen**	
Anti-PD-1 monotherapy (Pembrolizumab / Nivolumab)	8
Anti-CTLA-4 monotherapy (Ipilimumab)	14
Combination (Nivolumab + Ipilimumab or Pembrolizumab + Ipilimumab)	8
**Best Response**	
Complete Response (CR)	3
Partial Response (PR)	6
Stable Disease (SD)	2
Progressive Disease (PD)	16
Unknown / missing	3
**Clinical Benefit at 12 Months**	
Benefit	9
No Benefit	21
**Survival**	
Alive at last follow-up (Census OS = 0)	6
Deceased (Census OS = 1)	24
Median OS (months)	21

Supplementary Table S3: Immune checkpoints interaction cutoff values

Immune checkpoint interaction	QF-Pro scores (%) Cutoff values
PD-1/PD-L1	High >1.35%> Low
CTLA-4/CD80	High >1.25%> Low
TIGIT/CD155	High >2.41%> Low
LAG-3/MHC-II	High >0.96%> Low

Supplementary Table S4: Melanoma cohort clinical data analysis per treatment regimen

	anti-PD-1	anti-CTLA-4	anti-PD-1/CTLA-4 combination
Benefit at 12 months-patients with benefit/total (%)	n = 3/8 (37%)	n = 2/13 (13%)	n = 5/8 (62%)
Median DoR (months)	11	4	19
Median OS (months)	41	8	49
Metastatic patients/total (%)	n = 3/8 (37%)	n = 10/14 (71%)	n = 5/8 (62%)
